# Individual-Level Digital Determinants of Health and Technology Acceptance of Patient Portals: Cross-Sectional Assessment

**DOI:** 10.2196/56493

**Published:** 2024-06-10

**Authors:** Lindsey M Philpot, Priya Ramar, Daniel L Roellinger, Jane W Njeru, Jon O Ebbert

**Affiliations:** 1 Department of Medicine Mayo Clinic Rochester, MN United States; 2 Department of Quantitative Health Sciences Mayo Clinic Rochester, MN United States

**Keywords:** electronic health records, digital determinants of health, patient portals, eHealth, digital health, technology acceptance model, digital health literacy, digital inclusion, mobile phone

## Abstract

**Background:**

Digital determinants of health (DDoH), including access to technological tools and digital health literacy, function independently as barriers to health. Assessment for DDoH is not routine within most health care systems, although addressing DDoH could help mitigate differential health outcomes and the digital divide.

**Objective:**

This study aims to assess the role of individual-level DDoH factors on patient enrollment in and use of the patient portal.

**Methods:**

We developed a multimodal, cross-sectional survey and deployed it to 11,424 individuals based on their preferred mode and language documented within the electronic medical record. Based on the Technology Acceptance Model, enrollment in and intent to use the patient portal were the outcomes of interest. Perceived usefulness and ease of use were assessed to determine construct validity, and exploratory investigations included individual-level DDoH, including internet and device access, availability of technological support, medical complexity, individual relationship with the health care system, and digital health literacy. Counts (n) and proportions (%) were used to describe response categories, and adjusted and unadjusted odds ratios are reported.

**Results:**

This study included 1850 respondents (11,424 invited, 16.2% response rate), who were mostly female (1048/1850, 56.6%) and White (1240/1850, 67%), with an average age of 63 years. In the validation of the Technology Acceptance Model, measures of perceived ease of use (ie, using the patient portal will require a lot of mental effort; the patient portal will be very easy to use) and perceived usefulness (ie, the usefulness of the patient portal to send and receive messages with providers, schedule appointments, and refill medications) were positively associated with both enrollment in and intent to use the patient portal. Within adjusted models, perceived ease of use and perceived usefulness constructs, in addition to constructs of digital health literacy, knowing what health resources are available on the internet (adjusted odds ratio [aOR] 3.5, 95% CI 1.8-6.6), portal ease of use (aOR 2.8, 95% CI 1.6-5), and portal usefulness (aOR 2.4, 95% CI 1.4-4.2) were significantly associated with patient portal enrollment. Other factors associated with patient portal enrollment and intent to use included being comfortable reading and speaking English, reported use of the internet to surf the web or to send or receive emails, home internet access, and access to technology devices (computer, tablet, smartphone, etc).

**Conclusions:**

Assessing for and addressing individual-level DDoH, including digital health literacy, access to digital tools and technologies, and support of the relational aspects between patients, social support systems, and health care providers, could help mitigate disparities in health. By focusing efforts to assess for and address individual-level DDoH, an opportunity exists to improve digitally driven health care delivery outcomes like access and structural outcomes like bias built within algorithms created with incomplete representation across communities.

## Introduction

Digital health, or technology-based tools and services created to support the health and well-being of individuals, has the potential to improve and complement traditional models of health care [1]. The 2009 HITECH (Health Information Technology for Economic and Clinical Health) Act incentivized the electronic medical record (EMR) transition by health care systems, and over 95% of US hospitals currently use EMRs in the delivery of health care [2]. More recent progress under the 2020-2025 Federal Health Information Technology Strategic Plan enables patients access to their health information, encouraging many health care systems to facilitate patient access to personal health information as well as access to a “patient portal” through which health care seekers can send and receive care messages, schedule services, and manage medication prescriptions and medical devices. Research to date has been mixed on the impact of patient portal use on patient-centered clinical outcomes, but more promising for patient involvement in health care, improvement in disease-related knowledge, and lower health care use among users of patient portals. Significant disparities exist in the access to and use of digital health tools [3], limiting their use among certain populations [4]. Given the potential to improve these aspects of health and health care, the design of and access to digital health tools should consider ways to improve health outcomes while mitigating or reducing health disparities [5].

Digital determinants of health (DDoH), including access to technological tools, digital health literacy, and internet access, function independently as barriers to health, and a lack of these aspects negatively impacts health outcomes and patient experiences [6,7]. Data from the COVID-19 and Chronic Conditions cohort analyzing portal use suggested that the restrictive phase of the epidemic widened disparities in health literacy [8]. Analysis of the Health Information National Trends Survey demonstrated that minority populations had less access to an electronic health record compared to White and non-Hispanic populations [9]. In an assessment of patients admitted to a general medicine service, low eHealth literacy was associated with less awareness, use, and perceived usefulness of portals [10]; however, further investigation of the role of eHealth literacy and DDoH in a nationwide, multilanguage survey is needed. Assessment of enablers and barriers across health care–seeking populations may serve to address extant digital health disparities and mitigate disparity expansion that may occur with public health emergencies [11]. To enhance patient engagement with digital health technologies and solutions, assessment of individual factors of DDoH, including digital health literacy, has been purported to be the first step to enabling access to these digitized forms of health care [12].

DDoH are hypothesized to constitute several levels of factors, which can be translated into relevant individual, health care system, and policy interventions to mitigate and reduce disparities in health. At the individual level of DDoH, factors related to the practical use of technology, including reliable internet and device access; medical factors, including medical complexity and symptom burden of the individual; and relational factors of the individual with the health care provider or delivery system may be associated with individual ability and willingness to engage with digital health solutions [5,13]. Additionally, digital literacy, defined as the ability to access, evaluate, and judge information derived from electronic services, and health literacy, defined as the confidence and ability to apply derived knowledge to address health concerns [14], have merged to encompass digital health literacy [15]. Digital health literacy has been positively associated with access to technology and health literacy [16]. The technology acceptance model (TAM) is one of the most widely recognized theoretical models used to understand the perceptions of intended users of a new technology related to acceptance and use of that new technology. The TAM was built on the theory of reasoned action [17] and theory of planned behavior [18], which were based upon the information integration theory [19]. Under the TAM, an individual’s actual use of a technology is affected by their behavioral intention to use it [20]. Current applications of the TAM do not acknowledge the role of digital health literacy or other measures of DDoH as key components of the acceptance and the use of digital health technologies. The impact of internet access, medical complexity, and social support on use and intent to use a patient portal has not been completely evaluated. As opportunities for patient engagement in digital health care experience expand, little information exists on the extent to which digital health literacy acts as a barrier to patient engagement with a patient portal.

We undertook this study to validate perceived usefulness and ease of use among a large sample of patients engaged with a multistate, multisite health care system under the guidance of the TAM. We also investigated the impact of additional individual-level DDoH, including practical facilitators and barriers to internet and device access, medical complexity and symptom burden facilitators and barriers to use of the patient portal, individual relationships with the health care system and providers referring to the patient portal, and the role of digital health literacy as an individual factor associated with intent to use and use behavior of the patient portal. We hypothesize that increasing levels of digital health literacy will be positively associated with the intent to use and use behavior of the patient portal. We hypothesize that additional DDoH factors, including practical, medical, and relational individual-level attributes, will be associated with intent to use and use of the patient portal. We deployed a cross-sectional, multimodal, multilanguage survey to patients across our health care system to address the aims and hypotheses of this study.

## Methods

### Overview

We developed and deployed a cross-sectional survey through 2 modes to address the study aims, an electronic survey delivered to patient-provided email addresses and a paper survey delivered through US mail to patient-provided addresses of permanent residence. Deployment mode was selected based on patient-provided preferences for communications documented in the EMR. Electronic surveys were designed, managed, and deployed using Qualtrics survey software (Provo; Qualtrics) and included 3 total invitations to complete the survey instrument. Paper surveys were created using InDesign software (Microsoft Corporation) and deployed in a scannable booklet format in a single distribution wave. Stamped return envelopes were included with all mailed paper surveys. Those who did not respond to their electronic survey received a paper survey. Responses to electronic and paper surveys were appended into a single data set for analysis purposes. The build, deployment, and management of survey instruments were performed by the Mayo Clinic Survey Research Center.

### Instrument Development

**Figure 1:**
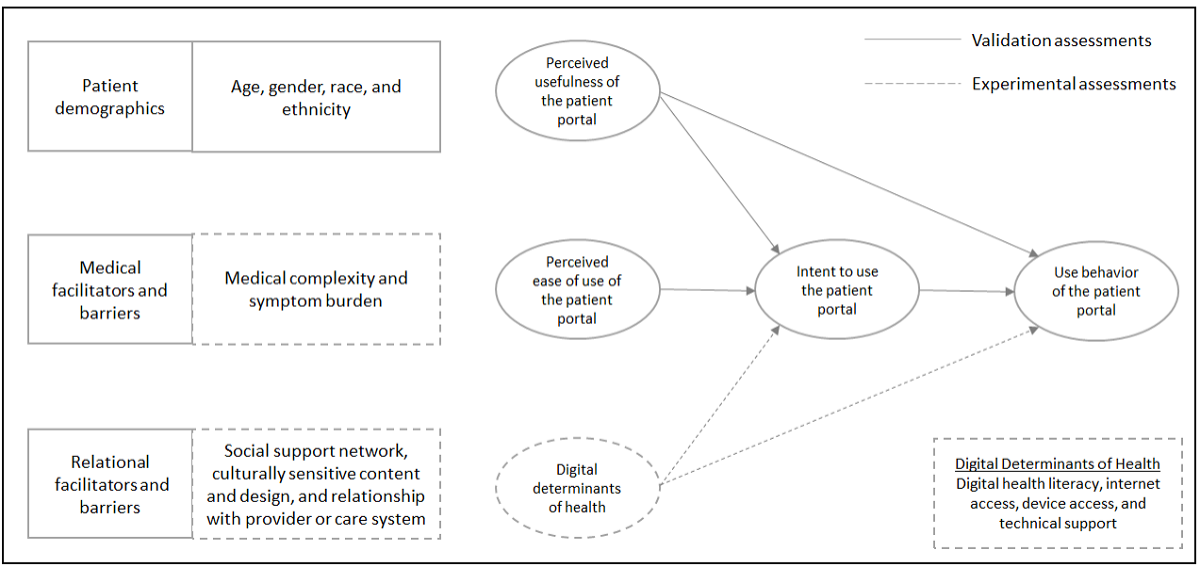
Theoretical relationships explored among patients as end users of the electronic medical record patient portal, based on the Technology Acceptance Model (TAM) and the proposed role of digital health literacy in a language-concordant, anonymous written and digital survey of 1850 patient portal users who received care at a large academic institution.

The theoretical model underpinning our investigation is depicted in Figure 1. The survey instrument was developed by an interdisciplinary team including physicians with expertise in digital health care and health disparities research, a scientist with expertise in survey design and methodology, a tenured analyst with experience in survey analysis and advanced statistics, and 2 analysts with experience in data management, survey analysis, and reporting. A literature review was performed using PubMed, Embase, and Google Scholar search engines to identify survey instruments and items addressing the survey domains of interest. A complete list of our survey items mapped to source tools and instruments is provided in Multimedia Appendix 1 [21-28]. Investigation domains where no survey instruments or items could be identified to adequately address the domain were drafted by the main study author (LMP), who has advanced training and experience in survey methodology. Survey instruments and individual survey items were consolidated into a single document for pilot testing and adjustment, as well as assessment for content validity. The survey instrument underwent 3 rounds of iterative piloting and adjustment by patient volunteers as well as our translation service provider (Morningside Translations LLC). Further information on the deployed survey instrument is included in the “Measures” section. Internal consistency, as a measure of instrument reliability, was assessed using Cronbach α on 5 variables: perceived usefulness (0.85=good), perceived ease of use (0.71=good), digital health literacy (0.91=excellent), intent to use (0.85=good), and use behavior (0.65=acceptable). Construct validation was performed by determining whether the survey instrument constructs performed similarly to those found within the TAM model, as indicated by the solid line associations in Figure 1. These approaches to reporting the reliability and validity of a survey instrument have been used previously in relation to the TAM [29]. Copies of the survey instruments deployed are included in Multimedia Appendices 2 and 3.

### Study Population

As part of the standard of care delivered at any Mayo Clinic site, patients are encouraged to register for an Epic patient portal that allows them to access their health information, upcoming and past medical appointments, and the ability to message with their care team, among other features. Since the institutional adoption of Epic in 2018, over 1 million patients have been invited to register for the patient portal, with nearly 90% of patients choosing to enroll. The remaining 10% of patients have registered but are inactive due to infrequent use, pending activation by the end user, or declination. Previous studies have reported that nonadopters of patient portals and digital health solutions respond to inquiries from health care provider-initiated surveys at half the rate of those who do participate in these services. We oversampled at a 1:2 ratio of active patient portal users to patients who declined to enroll, after limiting to patients who have had at least one health care interaction with Mayo Clinic in the 12 months before sampling. Patients were sampled if they were older than 18 years, not listed in the “do not contact” list, still living according to our internal records, and selected English or Spanish as their primary spoken language.

### Measures

The survey instrument contained validated survey instruments, individual items from published survey instruments, and novel survey items to address the study aims. A complete list of our survey items mapped to source tools and instruments is provided in Multimedia Appendix 1.

Perceived usefulness was defined by positive responses to the usefulness of any of the described patient portal functions (ie, message provider, schedule appointments, refills, access information, check symptoms, and see bills). Perceived ease of use was defined by agreement responses to the item “Overall, I believe that the patient portal will be easy to use” or disagreement responses to items indicating that the patient portal would be frustrating or require significant mental effort [21]. Digital health literacy items were measured independently as defined by Norman and Skinner [22] and included positive responses to knowing what health resources are available on the internet; where and how to find, evaluate, and use health resources on the internet; how to use the internet to answer questions about health; distinguishing high-quality health resources from low-quality health resources on the internet; and feeling confident in using information from the internet to make health decisions. Intent to use the patient portal was defined by agreement responses to items indicating intention to use the patient portal for any of the described patient portal items (ie, communicate with my provider, schedule an appointment with my provider, refill a prescription, access my health information, review the results of my tests, review education related to my health, and some other reason). Use behavior (enrollment in the patient portal) was defined by current patient portal access or “ever” access to the patient portal [23].

We also included survey item measures for demographics (ie, age, gender, race, comfort speaking and reading English), practical digital facilitators and barriers (ie, access to the internet, devices, connectivity, and use) [24], medical facilitators and barriers measured using the EQ-5D index [25], and relational facilitators and barriers as measured by social support for patient portal use and relationship with a health care provider or providers. Age was grouped into 4 categories based on previous literature indicating differing technology literacy and use patterns among age groups (<50 years, 50-65 years, 66-80 years, and >80 years) [30]. The final survey instrument, drafted in English, underwent translation into Spanish with linguistic validation by a third-party translation service provider (Morningside Translations LLC).

### Statistical Analysis

Survey responses were aggregated across modalities and languages. The analysis of differences in nonresponse was assessed by age, race, and gender. Responders who completed at least 90% of the survey questions were included in the analyses. Unadjusted and adjusted logistic regression models were used to investigate associations between exposure variables and facilitator and barrier variables with outcome variables. Associations between digital literacy alone and enrollment and intention to use the patient portal were also assessed. Factors that showed a significant association with outcomes independently (*P*<.05) were included in multivariable models. Results are presented as odds ratios with 95% CIs and *P* values, per guidelines for cross-sectional assessments [31]. Statistically significant associations were defined by 95% CIs that did not span the null and *P* values <.05. Descriptive comparisons of characteristics among responders and nonresponders were done using chi-square for gender and race and the 2-tailed *t* test for age, and results are provided in Multimedia Appendix 4. All analyses were conducted using SAS software (version 9.4; SAS Institute Inc).

### Ethical Considerations

This study (IRB#22-008356; principal investigator: LMP) received expedited review procedures by the Mayo Clinic Institutional Review Board and was approved as exempt from the requirement for the institutional review board approval (45 CFR 46.104d, Category 2). As protected health information was not being requested from survey respondents, HIPAA (Health Insurance Portability and Accountability Act) authorization was not required in accordance with 45 CFR 160.103. Study data were anonymous and deidentified. No compensation was provided to survey respondents.

## Results

### Overview

In total, 11,424 patients were eligible and sampled to receive an invitation to complete the survey. Of these, 1850 (16.2%) responded and completed at least 90% of the survey questions. Nonresponse analysis found significant differences in race and age between responders and nonresponders (Multimedia Appendix 4). More than 97% (1795/1850) of respondents reported having access to the internet from home; therefore, this question was removed as a factor in analyses. Of those who responded, the majority were female (1048/1850, 57.3%) and White (1240/1850, 67.7%), and had an average age of 63 years (Table 1). Most patients also reported being comfortable with reading English (1666/1850, 90.5%) and speaking English (1655/1850, 89.4%).

**Table 1:** Characteristics of respondents in a language-concordant, anonymous written, and digital survey of 1850 patient portal users who received care at a large academic institution.

Characteristics		Total, n (%)
**Age (years)**		
	<50	337 (18.8)
	50-65	552 (30.7)
	66-80	730 (40.6)
	>80	178 (9.9)
**Race**		
	White American or White European	1240 (67.7)
	Mexican or Mexican American	108 (5.9)
	Asian, South Asian, or Asian Pacific	95 (5.2)
	Black, African, or African American	85 (4.6)
	Mixed races	83 (4.5)
	None of listed	71 (3.9)
	Central or South American	51 (2.8)
	No answer	49 (2.7)
	Native American or Hawaiian or Pacific Islander	43 (2.3)
	Middle Eastern	6 (0.3)
**Gender**		
	Female	1048 (57.3)
	Male	747 (40.8)
	Choose not to answer	20 (1.1)
	Nonbinary	7 (0.4)
	Other	6 (0.3)
	Transgender female	2 (0.1)
**Level of comfort reading English**		
	Comfortable	1666 (90.5)
	Neutral or uncomfortable	162 (8.8)
**Level of comfort speaking English**		
	Comfortable	1655 (89.4)
	Neutral or uncomfortable	178 (9.6)

### Confirmation of the TAM: Perceived Portal Ease of Use and Usefulness on Portal Enrollment and Intent to Use

In confirmation of the TAM within our population, we observed that measures of perceived ease of use (ie, portal requiring mental effort and portal easy to use) and perceived usefulness (ie, usefulness of the portal in messaging with providers, scheduling appointments, and refilling medications) were positively associated with both enrollment in and intent to use the patient portal (Table 2).

**Table 2:** Univariable associations of respondent Technology Acceptance Model constructs by enrollment in and intent to use patient portals in a language-concordant, anonymous written and digital survey of 1850 patient portal users who received care at a large academic institution.

Patient factors		Enrolled in patient portal, OR^a^ (95% CI)	Intent to use patient portal, OR (95% CI)
**Age (years)**			
	50-65 versus <50	0.8 (0.5-1.6)	0.8 (0.5-1.4)
	66-80 versus <50	0.5 (0.3-0.8)^b^	0.4 (0.3-0.7)^b^
	>80 versus <50	0.2 (0.1-0.3)^b^	0.2 (0.1-0.3)^b^
Race or ethnicity		—^c,d^	—
Gender		—	—
**Comfort regarding English**			
	Comfortable reading English	4.1 (2.8-6.2)^b^	2.4 (1.6-3.7)^b^
	Comfortable speaking English	3.5 (2.3-5.2)^b^	2 (1.3-3.1)^c^
**Perceived ease of use**			
	Portal use requires a lot of mental effort (disagree)	4.2 (2.8-6.2)^b^	6.6 (4.6-9.3)^b^
	Portal use is frustrating (disagree)	3.8 (2.6-5.5)^b^	6.8 (4.9-9.5)^b^
	Portal will be easy to use	6.9 (4.7-10.1)^b^	14.5 (9.5-22)^b^
**Perceived usefulness**			
	Internet is useful for making decisions about health or accessing health resources on the internet is important	5.2 (3.7-7.4)^b^	8.5 (6-11.9)^b^
	My patient portal is useful to message provider, schedule appointments, refills, access information, check symptoms, or see bills	5.3 (3.7-7.6)^b^	10 (7.1-14.1)^b^
Medical complexity and symptom burden (EQ-5D index)		2.2 (1.2-4.1)^c^	3.4 (2-5.8)^b^
**Relational facilitators and barriers**			
	I have someone who encourages me to seek medical assistance through the patient portal.	1.8 (1.2-2.7)^c^	2.7 (1.8-4)^b^
	Medical provider has asked about my cultural or spiritual beliefs related to health.	1.3 (0.8-2.1)	1.7 (1.1-2.7)^c^
	I have a good relationship with those who I receive health care from and I feel my health care provider has my best interests at heart.	1.6 (1.1-2.4)^c^	2.3 (1.6-3.2)^b^
	I have someone I can turn to if I need help accessing the patient portal.	2 (1.4-2.8)^b^	2.9 (2.1-4)^b^
**Digital determinants of health**			
	Connect to the internet to surf the web or to send and receive emails	13.5 (8.7-21.1)^b^	11.5 (7.5-17.8)^b^
	Access to device (computer, tablet, smartphone, etc)	15.7 (6.5-37.8)^b^	24.4 (9.3-63.7)^b^
	Access the internet from home	15.8 (8.9-27.9)^b^	17.7 (9.9-31.6)^b^
	Satisfied with ability to access the internet	3.8 (2.5-5.5)^b^	6 (4.2-8.5)^b^
**Digital health literacy**			
	I know what health resources are available on the internet.	5.4 (3.8-7.7)^b^	4.5 (3.3-6.2)^b^
	I know where to find helpful health resources on the internet.	5.9 (4.2-8.4)^b^	6 (4.4-8.3)^b^
	I know how to find helpful health resources on the internet.	4.8 (3.4-6.7)^b^	6.2 (4.5-8.6)^b^
	I know how to use the internet to answer my questions about health.	5.4 (3.8-7.6)^b^	6.7 (4.9-9.3)^b^
	I know how to use the health information I find on the internet to help me.	4.5 (3.2-6.4)^b^	5.7 (4.1-7.9)^b^
	I have the skills I need to evaluate the health resources I find on the internet.	3.5 (2.5-5)^b^	4.9 (3.5-6.8)^b^
	I can tell high-quality health resources from low-quality health resources on the internet.	3.9 (2.7-5.8)^b^	4.7 (3.3-6.7)^b^
	I feel confident in using information from the internet to make health decisions.	3.5 (2.4-5.1)^b^	4.5 (3.1-6.5)^b^

^a^OR: odds ratio.

^b^*P* value <.001.

^c^*P* value <.05.

^d^Not applicable.

### DDoH on Portal Enrollment and Intent to Use

**Figure 2:**
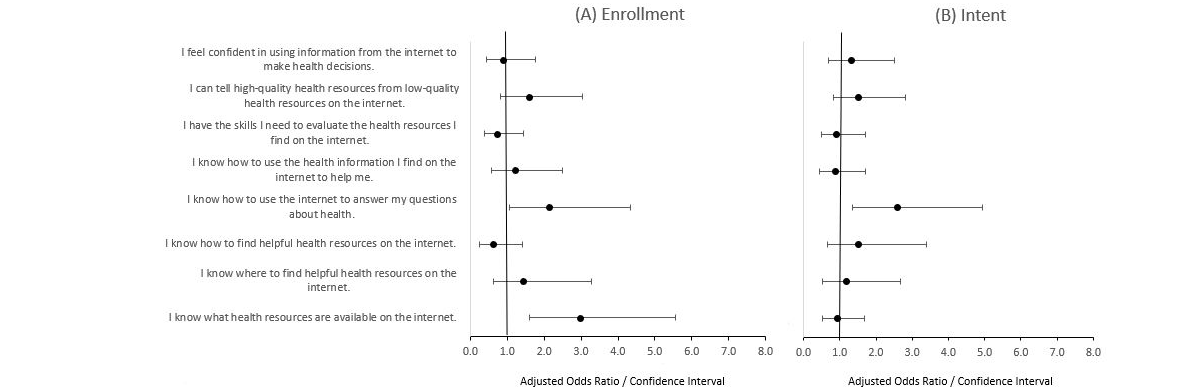
Multivariable role of digital health literacy on patient enrollment in portal (A) and digital health literacy on patient intent to use portal (B) in a language-concordant, anonymous written and digital survey of 1850 patient portal users who received care at a large academic institution.

**Figure 3:**
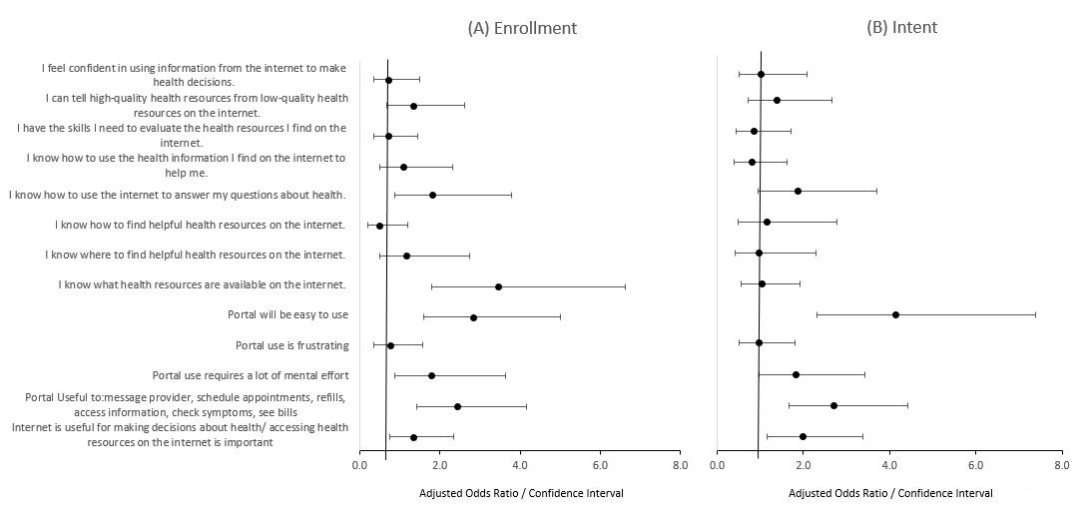
Multivariable role of digital health literacy on patient enrollment in the portal (A) and digital health literacy on patient intent to use the portal (B) including ease of portal use and portal usefulness constructs, in a language-concordant, anonymous written and digital survey of 1850 patient portal users who received care at a large academic institution.

Within our univariable assessments, we found that all measures of digital health literacy were positively associated with both enrollment in and intent to use the patient portal (Table 2). The largest uncontrolled measures of effect were noted for knowing how to use the internet to answer questions about health, knowing where to find helpful health resources on the internet, and knowing how to use health information found on the internet. Within our multivariable models constructs of digital health literacy on patient enrollment in the portal, we observed that knowing how to use the internet to answer health-related questions (adjusted odds ratio [aOR] 2.1, 95% CI 1.1-4.3) and knowing what health resources are available on the internet (aOR 3, 95% CI 1.6-5.6) were associated with portal enrollment, and knowing how to use the internet to answer health-related questions was associated with intent to use the patient portal (aOR 2.6, 95% CI 1.4-4.9; Figures 2A and B). When including all perceived ease of use and usefulness constructs in addition to constructs of digital health literacy, knowing what health resources are available on the internet (aOR 3.5, 95% CI 1.8-6.6), perceived ease of use (aOR 2.8, 95% CI 1.6-5), and usefulness (aOR 2.4, 95% CI 1.4-4.2) were significantly associated with patient portal enrollment (Figure 3A). Perceived ease of use (aOR 4.1, 95% CI 2.3-7.4), usefulness (aOR 2.7, 95% CI 1.7-4.4), and perceiving that the internet is useful for making health decisions (aOR 2, 95% CI 1.2-3.4) were significantly associated with intent to use the patient portal in the final multivariable models (Figure 3B).

### Patient Demographics, Medical Facilitators and Barriers, Relational Facilitators and Barrers on Portal Enrollment, and the Intent to Use

Within our univariable assessments, patients who were older had significantly lower odds of enrollment in and intent to use a patient portal (Table 2). Additionally, those who were comfortable reading and speaking English had significantly higher odds of enrolling in and intending to use a patient portal. We also observed that other DDoH, such as use of the internet to surf the web or to send or receive emails, home internet access, and access to technology devices (computer, tablet, smartphone, etc) were positively associated with both enrollment in and intent to use the patient portal. Additionally, we observed significant relationships between patients with increasing medical complexity (EQ-5D) and both enrollment in and intent to use the patient portal. Most of the relational facilitators and barriers that we assessed were also significant with enrollment in and intent to use the patient portal, including having someone who encourages the patient to seek medical assistance through the portal, having a good relationship with health care providers, and having someone to turn to when help is needed accessing the patient portal. Reporting that medical providers have asked about cultural or spiritual beliefs was associated with increased odds of intent to use the patient portal but was not significantly associated with portal enrollment.

## Discussion

### Overview

We undertook this study to validate perceived usefulness and ease of use among a large sample of patients engaged with a multistate, multisite health care system under the guidance of the TAM. Additionally, we sought to explore the impact of individual-level DDoH on use and intent to use a patient portal. Investigative factors included practical facilitators and barriers to internet and device access, technological support, medical complexity and symptom burden, individual relationships with the health care system and providers referring to the patient portal, social encouragement and support to use tools like the patient portal, and digital health literacy. We hypothesized that increasing levels of digital health literacy would be associated with patient intent to use the patient portal and patient use behavior of the patient portal. We additionally hypothesized that additional DDoH factors, including practical, medical, and relational individual-level patient attributes, would be associated with intent to use and use of the patient portal.

We observed that digital health literacy has a significant role to play in enabling patients to engage with digital health tools. Health literacy has previously been associated with patients’ ability to access and understand clinical lab test results [32] and to access patient-facing medical records within Sweden [33] and the United Kingdom [34]. Within the United States, low digital health literacy has been associated with lower awareness, lower use, and lower perceived usefulness of the patient portal among a small sample of hospital inpatients [10]. Research shows that interest and engagement in digital health tools have increased since the COVID-19 pandemic [8,35]. We report the largest cross-sectional study performed to assess the role of digital health literacy on intent to use and use behavior of patient portal tools since the observed increase in digital engagement after the COVID-19 pandemic, validating that digital health literacy remains a key component in the use of patient portals among patients in the United States.

We observed that other patient factors, including being comfortable reading and speaking English, reported use of the internet to surf the web or to send or receive emails, having at-home internet access, and access to technology devices (computer, tablet, smartphone, etc), were positively associated with both enrollment in and intent to use the patient portal. Research since 2020 has included differential use of self-scheduling aspects of the patient portal by race, ethnicity, and primary spoken language [36], and differential use by patients with chronic health conditions by age, gender, race, and ethnicity [8]. These individual-level DDoH factors were consistently observed within this study and corroborate those reported previously.

We observed that individual-level relational factors were associated with both the intent to use and the use behavior of the patient portal. We found that individuals who reported having a social support structure encouraging and facilitating portal use were more likely to do so. This finding is a new contribution to the literature, although social support and encouragement have been recognized as facilitators previously within the TAM under the constructs of social influence and perceived norms within communities. We also observed that having a good relationship with one’s health care provider was associated with increased odds of use of the patient portal, a finding that has been demonstrated previously among a nationally representative sample of adult patients with cancer [37].

DDoH and social determinants of health are associated with disparities in health experienced across different communities. Assessing for DDoH can help health care systems target interventions that address disparities in access to digital tools and technologies that are becoming increasingly used by health care systems, provider teams, and patients. DDoH are directly tied to health care delivery outcomes [5], like access to services, appointments, and care team members that are evolving to incorporate more self-service scheduling and messaging options. DDoH are tied to patient, caregiver, and community experiences of health care [5], as provider groups create digital education content and care plans intended to help support patients. Finally, DDoH are tied to the evolution of algorithms that are being created to help identify patient needs, align needs with care resources, and facilitate connections with community resources intended to serve people. If we do not take steps to create a digitally inclusive environment by assessing and addressing individual factors of DDoH, we are at risk of exacerbating algorithmic bias in future care models [5].

Our findings are consistent with previous literature observing that individuals with limited health-related literacy, limited experience with technology and computers, and adults of older age experience barriers using patient portals. Our findings underscore the need for health care systems to facilitate the use of patient portals by addressing barriers to their use, providing access to individuals to help patients experiencing difficulties, and offering language-congruent portal services. Our findings also highlight the need for health care systems to create education and tools that can help patients navigate through electronic health sources, further enabling them to be partners in their health care.

Our investigation had several limitations. We observed differential response rates to both our email-based survey based on race, age, and primary language spoken. We deployed multiple strategies to reduce nonresponse bias, including the formal translation of our entire survey instrument, an invitation letter, and introductory comments by a third-party vendor who specializes in language translation. We also pilot-tested our survey instrument with both English- and Spanish-speaking individuals to make the survey as easy-to-understand as possible. Finally, we used multiple contact methods, including a crossover design from electronic invitations to paper mail–based invitations to include the voices of as many individuals as possible. Our interpretability across results, particularly among those of non-White, younger, and Spanish-speaking individuals, may be limited.

This study also has several strengths, including the use of a recognized theoretical model (TAM) and the use of a digital health literacy assessment tool that was independently developed and published. We were also able to sample within a large national population and intentionally oversample individuals with traditionally lower response rates (patient portal nonusers and individuals who primarily speak Spanish responding to a primarily English-speaking organization).

### Conclusion

Assessing for and addressing individual-level DDoH, including digital health literacy, access to digital tools and technologies, and support of the relational aspects between patients, social support systems, and health care providers, could help mitigate and reduce disparities in health. By focusing efforts to assess for and address individual-level DDoH, we have an opportunity to improve digitally driven health care delivery outcomes like access and experience and structural outcomes like bias built within algorithms created with incomplete representation across communities.

## Appendix

Multimedia Appendix 1 Survey domains and items.

Multimedia Appendix 2 English version of deployed survey.

Multimedia Appendix 3 Spanish version of deployed survey.

Multimedia Appendix 4 Assessment for nonresponse bias.

## Abbreviations

aOR: adjusted odds ratio
DDoH: digital determinants of health
EMR: electronic medical record
HIPAA: Health Insurance Portability and Accountability Act
HITECH: Health Information Technology for Economic and Clinical Health
TAM: Technology Acceptance Model

## Data Availability

The data sets generated or analyzed during this study are available from the corresponding author upon reasonable request.
