# Supplementary material for: Individual-Level Digital Determinants of Health and Technology Acceptance of Patient Portals: Cross-Sectional Assessment
Source: JMIR Form Res. 2024 Jun 10;8:e56493. doi: 10.2196/56493 (PMC11196914; doi:10.2196/56493)
Supplement: Multimedia Appendix 1 [file formative_v8i1e56493_app1.docx]

| **Survey Domain** | **Survey Item** | **Reference** |
| --- | --- | --- |
| Screener | Have you ever been offered online access to your medical records by your health care provider? | HINTS Cycle 5 [25] |
| Digital Health Care Literacy | I can use application / programs (like Zoom) on my cell phone, computer, or another electronic device on my own (without asking for help from someone else). | Nelson, 2022 [35] |
| Digital Health Care Literacy | I can set up a video chat using my cell phone, computer, or another electronic device on my own (without asking for help form someone else). | Nelson, 2022 [35] |
| Digital Health Care Literacy | I can solve or figure out how to solve basic technical issues on my own (without asking for help from someone else). | Nelson, 2022 [35] |
| Digital Determinants of Health | eHeals Scale | Norman, 2006 [23] |
| Ease of Use | Using the patient portal will quire a lot of mental effort. | Emani 2018 [22] |
| Ease of Use | Learning to use the patient portal will be easy for me. | Emani 2018 [22] |
| Ease of Use | Using the patient portal will be frustrating. | Emani 2018 [22] |
| Ease of Use | Overall, I believe that the patient portal will be easy to use. | Emani 2018 [22] |
| Usefulness | How useful are the following elements in a patient portal? Send and receive messages with my provider(s). | NEW |
| Usefulness | How useful are the following elements in a patient portal? Ability to schedule appointments. | NEW |
| Usefulness | How useful are the following elements in a patient portal? Ability to refill my medication(s). | NEW |
| Usefulness | How useful are the following elements in a patient portal? Ability to access my information whenever I need / want. | NEW |
| Usefulness | How useful are the following elements in a patient portal? Ability to view the results of my tests. | NEW |
| Usefulness | How useful are the following elements in a patient portal? Ability to review my vaccination / immunization records. | NEW |
| Usefulness | How useful are the following elements in a patient portal? Ability to check my symptoms. | NEW |
| Usefulness | How useful are the following elements in a patient portal? Ability to see results of my COVID-19 testing. | NEW |
| Usefulness | How useful are the following elements in a patient portal? Ability to see my bills. | NEW |
| Experience with IT | Do you ever go on-line to access the Internet or World Wide Web, or to send and receive e-mail? | HINTS Cycle 5 [25] |
| Prior Use of Health IT | In the past 12 months, have you used a computer, smartphone, or other electronic means to look for health or medical information for yourself? | HINTS Cycle 5 [25] |
| Prior Use of Health IT | In the past 12 months, have you used a computer, smartphone, or other electronic means to look for health or medical information for someone else? | HINTS Cycle 5 [25] |
| Prior Use of Health IT | In the past 12 months, have you used a computer, smartphone, or other electronic means to buy medicine or vitamins online? | HINTS Cycle 5 [25] |
| Prior Use of Health IT | In the past 12 months, have you used a computer, smartphone, or other electronic means to look for a health care provider? | HINTS Cycle 5 [25] |
| Prior Use of Health IT | In the past 12 months, have you used a computer, smartphone, or other electronic means to use email or the internet to communicate with a doctor or doctor's office? | HINTS Cycle 5 [25] |
| Prior Use of Health IT | In the past 12 months, have you used a computer, smartphone, or other electronic means to make appointments with a health care provider? | HINTS Cycle 5 [25] |
| Prior Use of Health IT | In the past 12 months, have you used a computer, smartphone, or other electronic means to fill out forms or paperwork related to your health care? | HINTS Cycle 5 [25] |
| Prior Use of Health IT | In the past 12 months, have you used a computer, smartphone, or other electronic means to look up test results? | HINTS Cycle 5 [25] |
| Prior Use of Health IT | On your tablet or smartphone, do you have any software applications or apps related to health? | HINTS Cycle 5 [25] |
| Prior Use of Health IT | Has your tablet or smartphone helped you achieve a health-related goal such as quitting smoking, losing weight, or increasing physical activity? | HINTS Cycle 5 [25] |
| Prior Use of Health IT | Has your tablet or smartphone helped you in discussion with your healthcare provider? | HINTS Cycle 5 [25] |
| Prior Use of Health IT | Other than a tablet or smartphone, have you used an electronic device to monitor or track your health within the last 12 months? Examples include Fitbit, blood glucose meters, and blood pressure monitors. | HINTS Cycle 5 [25] |
| Practical Facilitator | When you use the Internet, do you access it through… A regular dial-up telephone line | HINTS Cycle 5 [25] |
| Practical Facilitator | When you use the Internet, do you access it through… Broadband such as DSL, cable, or FiOS | HINTS Cycle 5 [25] |
| Practical Facilitator | When you use the Internet, do you access it through… A cellular network (i.e., telephone, 3G/4G) | HINTS Cycle 5 [25] |
| Practical Facilitator | When you use the Internet, do you access it through… A wireless network (Wi-Fi) | HINTS Cycle 5 [25] |
| Practical Facilitator | Please indicate if you have a tablet computer like an iPad, Samsung Galaxy, Motorola Xoom, or Kindle Fire | HINTS Cycle 5 [25] |
| Practical Facilitator | Please indicate if you have a smartphone, such as an iPhone, Android, Blackberry, or Windows phone | HINTS Cycle 5 [25] |
| Practical Facilitator | Please indicate if you have a basic cell phone only | HINTS Cycle 5 [25] |
| Practical Facilitator | To what extent are you satisfied with your internet speed? | HINTS Cycle 5 [25] |
| Practical Facilitator | Why have you not accessed your medical records online? Is it because you prefer to speak to your health care provider directly? | HINTS Cycle 5 [25] |
| Practical Facilitator | Why have you not accessed your medical records online? Is it because you do not have a way to access the website? | HINTS Cycle 5 [25] |
| Practical Facilitator | Why have you not accessed your medical records online? Is it because you did not have a need to use your online medical record? | HINTS Cycle 5 [25] |
| Practical Facilitator | Why have you not accessed your medical records online? Is it because you were concerned about the privacy or security of the website that had your medical records? | HINTS Cycle 5 [25] |
| Practical Facilitator | Why have you not accessed your medical records online? Is it because you don’t have an online medical record? | HINTS Cycle 5 [25] |
| Practical Facilitator | Why have you not accessed your medical records online? Is it because…Other | HINTS Cycle 5 [25] |
| Innovativeness | If I heard about a new information technology, I would look for ways to experiment with it. | Emani 2018 [22] |
| Innovativeness | Among my peers, I am usually the first to try out new information technologies. | Emani 2018 [22] |
| Intention | I intend to use the patient poral the next time I need to communicate with my provider. | NEW |
| Intention | I intend to use the patient poral the next time I need to schedule an appointment with my provider. | NEW |
| Intention | I intend to use the patient portal the next time I need to refill my prescription(s). | NEW |
| Intention | I intend to use the patient portal the next time I need to access my health information. | NEW |
| Intention | I intend to use the patient portal the next time I need to review the results of my tests. | NEW |
| Intention | I intend to use the patient portal the next time I need to review education related to my health. | NEW |
| Intention - Pre intention | Please select the choice that most reflects your feeling on the patient portal: I've never heard of the patient portal. | Robb 2021 (Modified) [24] |
| Intention - Pre intention | Please select the choice that most reflects your feeling on the patient portal: I never thought about enrolling in the patient portal. | Robb 2021 (Modified) [24] |
| Intention - Pre intention | Please select the choice that most reflects your feeling on the patient portal: I'm undecided about enrolling in the patient portal. | Robb 2021 (Modified) [24] |
| Intention - Pre intention | Please select the choice that most reflects your feeling on the patient portal: I have enrolled in the patient portal. | Robb 2021 (Modified) [24] |
| Intention + Use Behavior | Please select the choice that most reflects your feeling on the patient portal: I routinely use the patient portal. | Robb 2021 (Modified) [24] |
| Intention + Use Behavior | Please select the choice that most reflects your feeling on the patient portal: I have used and plan to continue to use the patient portal. | Robb 2021 (Modified) [24] |
| Intention - Inaction | Please select the choice that most reflects your feeling on the patient portal: I decided not to enroll in the patient portal. | Robb 2021 (Modified) [24] |
| Intention - Importance | How important would you say it is for you to use the patient portal? (0 Not at all important - 10 Extremely important) | Miller 2002 [36] |
| Medical Facilitator | Mobility | EQ5D [26] |
| Medical Facilitator | Self-Care | EQ5D [26] |
| Medical Facilitator | Usual Activities | EQ5D [26] |
| Medical Facilitator | Pain / Discomfort | EQ5D [26] |
| Medical Facilitator | Anxiety / Depression | EQ5D [26] |
| Medical Facilitator | In general, would you say your health is: Excellent, Very good, Good, Fair, Poor | All of Us Participant Provided Information (2018) [37] |
| Medical Facilitator | In general, would you say your quality of life is: Excellent, Very good, Good, Fair, Poor | All of Us Participant Provided Information (2018) [37] |
| Medical Facilitator | In general, how would you rate your physical health? Excellent, Very good, Good, Fair, Poor | All of Us Participant Provided Information (2018) [37] |
| Medical Facilitator | In general, how would you rate your mental health, including your mood and your ability to think? | All of Us Participant Provided Information (2018) [37] |
| Medical Facilitator | In general, how would you rate your satisfaction with your social activities and relationships? | All of Us Participant Provided Information (2018) [37] |
| Medical Facilitator | In the past 7 days, how would you rate your fatigue? | All of Us Participant Provided Information (2018) [37] |
| Relational Facilitators | I have someone who encourages me to seek medical assistant through the patient portal. | NEW |
| Relational Facilitators | I have someone I can turn to if I need help accessing the patient portal. | NEW |
| Relational Facilitators | Do you have a person you identify as your primary care provider (doctor, nurse, nurse practioner, other) | NEW |
| Relational Facilitators | If yes, how long have you been seeing this person for your own health? (Less than 1 year, 1-5 years, more than 5 years) | NEW |
| Relational Facilitators | I have a good relationship with my primary care provider. | NEW |
| Relational Facilitators | I feel like my primary care provider has my best interests at heart. | NEW |
