# Supplementary material for: Individual-Level Digital Determinants of Health and Technology Acceptance of Patient Portals: Cross-Sectional Assessment
Source: JMIR Form Res. 2024 Jun 10;8:e56493. doi: 10.2196/56493 (PMC11196914; doi:10.2196/56493)
Supplement: Multimedia Appendix 2 [file formative_v8i1e56493_app2.pdf]

MAYO CLINIC

Digital Health Inclusion Survey

Place barcode label here.

MARKING INSTRUCTIONS

- **Use a No. 2 pencil or a blue or black ink pen only.**
- **Do not use pens with ink that soaks through the paper.**
- **Make solid marks that fill the response completely.**
- **Make no stray marks on this form.**

CORRECT: ⬬ **INCORRECT:** ✓ ✗

PLEASE PROVIDE

Your name (please print): ______________________

Today's date: __ __ – __ __ – 2 0 __ __

Month Day Year

PLEASE DO NOT WRITE IN THIS AREA

[SERIAL]

The following questions ask about your use of computers, desktop computers, smartphones and the internet.

1. Do you ever connect to the internet to surf the web or to send and receive emails?

- Yes
- No
- Don't know

2. Please indicate if you have access to the following devices: (Select all that apply.)

- A desktop or laptop computer
- A tablet like an iPad, Samsung Galaxy, Motorola Xoom, or Kindle Fire
- A smartphone, such as an iPhone, Android, Blackberry or Windows phone
- None of the above

3. Do you access the internet from home?

- Yes
- No
- Don't know

4. If yes, how satisfied are you with your ability to access the internet?

- Satisfied
- Neither satisfied or dissatisfied
- Dissatisfied

5. Please select Yes, No, or I don't know for the following statements.

	Yes	No	I don't know
When I buy a new electronic device, I usually need someone else to set it up or show me how to use it.	○	○	○
Among my peers, I am usually the first to try out new technologies. . . .	○	○	○
If I need to send a message to my doctor today, I would easily be able to do so.	○	○	○
I have used a tablet or smartphone to help me achieve a health-related goal such as quitting smoking, losing weight, or getting more physical activity.	○	○	○
I would be interested in learning about alternative/new ways to contact my doctor.	○	○	○

We would like to ask you for your opinion and about your experience using the internet for health information. For each statement, select which response best reflects your opinion and experience *right now.*

6. How <u>useful</u> do you feel the internet is in helping you in making decisions about your health?

- Not useful at all
- Not useful
- Unsure
- Useful
- Very useful

7. **How important is it for you to be able to access health resources on the internet?**

- Not important at all
- Not important
- Unsure
- Important
- Very important

8. **Please select Yes, No, or I don't know for the following statements.**

	Yes	No	I don't know
I know what health resources are available on the internet.			
I know where to find helpful health resources on the internet.			
I know how to find helpful health resources on the internet.			
I know how to use the internet to answer my questions about health.			
I know how to use the health information I find on the internet to help me.			
I have the skills I need to evaluate the health resources I find on the internet.			
I can tell high-quality health resources from low-quality health resources on the internet.			
I feel confident in using information from the internet to make health decisions.			

The following questions ask you about the Mayo Clinic patient portal. The patient portal is a tool that allows you access to Mayo Clinic resources and tools on a website or an application for your phone or tablet computer.

9. **Have you *ever* been offered online access to your medical records by your healthcare provider?**

- Yes
- No
- I don't know

10. **Have you ever *had* a Mayo Clinic patient portal account?**

- Yes
- No
- I don't know

11. Do you *currently* have access to a Mayo Clinic patient portal account?

○ Yes   ○ No   ○ I don't know

12. If yes, have you ever accessed your Mayo Clinic patient portal account?

○ Yes   ○ No   ○ I don't know

13. Have you stopped using the Mayo Clinic patient portal for any reason at all?

○ Yes   ○ No   ○ I don't know

14. Why have you stopped using the Mayo Clinic patient portal? (Select all that apply.)

○ I prefer to speak to my health care provider directly.
○ I do not have a way to access the website.
○ I do not have a need to use my online medical record.
○ I was concerned about the privacy or security of the website that had my medical records.
○ I experience difficulties in accessing my patient portal (passwords, devices, etc.).
○ Please list any other reasons you do not use a Mayo Clinic patient portal:

____

____

____

____

15. If no or I don't know, would you be interested in obtaining access to a Mayo Clinic patient portal account?

○ Yes   ○ No   ○ I don't know

16. Did you have a Mayo Clinic patient portal account at one time, but decided to stop using it?

○ Yes   ○ No   ○ I don't know

17. Why have you not used a Mayo Clinic patient portal? (Select all that apply.)

○ I prefer to speak to my health care provider directly.
○ I do not have a way to access the website.
○ I do not have a need to use my online medical record.
○ I was concerned about the privacy or security of the website that had my medical records.
○ I experience difficulties in accessing my patient portal (passwords, devices, etc.).
○ Please list any other reasons you do not use a Mayo Clinic patient portal:

____

____

____

____

18. How important would you say it is for you to use the patient portal?

○	○	○	○	○	○	○	○	○	○
1	2	3	4	5	6	7	8	9	10
Not at all important									Extremely important

19. The following questions ask you about how easy it is to use the patient portal. Please indicate your level of agreement with the following statements.1

	Agree	Neither agree nor disagree	Disagree
Using the patient portal will require a lot of mental effort.	○	○	○
Using the patient portal will be frustrating.	○	○	○
Overall, I believe that the patient portal will be very easy to use.	○	○	○

20. The following questions ask you about the support you have at home to use the patient portal. Please indicate your level of agreement with the following statements.1

	Agree	Neither agree nor disagree	Disagree
I have someone who encourages me to seek medical assistance through the patient portal.	⬭	⬭	⬭
I have someone I can turn to if I need help accessing the patient portal.	⬭	⬭	⬭

21. The following questions ask you about how useful you find the tools within the patient portal. How useful are the following elements in a patient portal?1

	Not useful at all	Not useful	Unsure	Useful	Very Useful
Send and receive messages with my provider(s).	⬭	⬭	⬭	⬭	⬭
Ability to schedule appointments.	⬭	⬭	⬭	⬭	⬭
Ability to refill my medication(s).	⬭	⬭	⬭	⬭	⬭
Ability to access my information whenever I need/want.	⬭	⬭	⬭	⬭	⬭
Ability to view the results of my tests.	⬭	⬭	⬭	⬭	⬭
Ability to review my vaccination/immunization records.	⬭	⬭	⬭	⬭	⬭
Ability to check my symptoms.	⬭	⬭	⬭	⬭	⬭
Ability to see my bills.	⬭	⬭	⬭	⬭	⬭

22. The following questions ask you about your intent on using the Mayo Clinic patient portal in the future. Please indicate your level of agreement with the following statements.

	Agree	Neither agree nor disagree	Disagree
I intend to use the patient portal the next time I need to communicate with my provider.	⬭	⬭	⬭
I intend to use the patient portal the next time I need to schedule an appointment with my provider.	⬭	⬭	⬭
I intend to use the patient portal the next time I need to refill a prescription.	⬭	⬭	⬭
I intend to use the patient portal the next time I need to access my health information.	⬭	⬭	⬭
I intend to use the patient portal the next time I need to review the results of my tests.	⬭	⬭	⬭
I intend to use the patient portal the next time I need to review education related to my health.	⬭	⬭	⬭
I intend to use the patient portal for some other reason.	⬭	⬭	⬭

Under each heading, please select the ONE response that best describes your health TODAY.

23. Mobility2

- I have no problems walking
- I have slight problems walking
- I have moderate problems walking
- I have severe problems walking
- I am unable to walk

24. Self-care2

- I have no problems washing or dressing myself
- I have slight problems washing or dressing myself
- I have moderate problems washing or dressing myself
- I have severe problems washing or dressing myself
- I am unable to wash or dress myself

25. Usual Activities (e.g., work, study, housework, family or lesiure activities)2

- I have no problems doing my usual activities
- I have slight problems doing my usual activities
- I have moderate problems doing my usual activities
- I have severe problems doing my usual activities
- I am unable to do my usual activities

26. Pain/Discomfort2

- I have no pain or discomfort
- I have slight pain or discomfort
- I have moderate pain or discomfort
- I have severe pain or discomfort
- I have extreme pain or discomfort

27. Anxiety/Depression2

- I am not anxious or depressed
- I am slightly anxious or depressed
- I am moderately anxious or depressed
- I am severly anxious or depressed
- I am extremely anxious or depressed

28. We would like to know how good or bad your health is TODAY. This scale is numbered from 0-100. 100 means the best health you can imagine. 0 means the worst health you can imagine. Mark an X on the scale to indicate how your health is TODAY. Now, please write the number you marked the scale in the box below.

Your health today= [| |]

The worst health you can imagine — The best health you can imagine

0 5 10 15 20 25 30 35 40 45 50 55 60 65 70 75 80 85 90 95 100

29. In general, would you say your health is:

- Excellent
- Very good
- Good
- Fair
- Poor

30. I have a good relationship with those who I receive health care from.

- Agree
- Neither agree nor disagree
- Disagree

31. I feel like my healthcare provider has my best interests at heart.

- Agree
- Neither agree nor disagree
- Disagree

PLEASE DO NOT WRITE IN THIS AREA

[SERIAL]

32. Has a doctor, nurse nurse practitioner, or other medical person ever asked you about your cultural or spiritual beliefs related to your health?

- Yes
- No
- I don't know

Demographics

33. Which gender identity do you most closely identify?

- Female
- Male
- Transgender Female
- Transgender Male
- Non-binary/gender nonconforming
- Prefer not to answer
- Identity not listed, please specify: ______________________

34. Which race(s) do you most closely identify? (Select all that apply.)

- Asian, South Asian, or Asian Pacific
- Black, African, or African American
- Central or South American
- Mexican or Mexican American
- Middle Eastern
- Native American
- Native Hawaiian or Pacific Islander
- White American, Caucasian, or White European
- Mixed race(s)
- None of these
- Prefer not to answer
- Other race not listed, please specify: ______________________

35. Which language(s) are you most comfortable reading? (Select all that apply.)

- Arabic
- Cantonese
- English
- Farsi
- Hindi
- Mandarin
- Spanish
- Somali
- Urdu
- Prefer not to answer
- Other language not listed, please specify: ______________________

36. How comfortable would you say you are <u>reading</u> in English?

- Uncomfortable
- Neutral
- Comfortable

37. How comfortable would you say you are <u>speaking</u> in English?

- Uncomfortable
- Neutral
- Comfortable

38. Please let the research team know of any other thoughts or comments you have related to this topic:

____

____

____

Thank you for completing this survey!

Questions 13-15: Emani S, Peters E, Karson AS, Lipsitz SR, LaRocca R, Stone J, Suric V, Wald JS, Wheeler A, Williams DH, Bates DW. Who adopts a patient portal?: An application of the diffusion of innovation model. J Innov Health Inform. 2018 Oct 25;25(3); 149-157. doi: 10.14236/jhi.v25i3.991. PMID: 30398458.

Questions 17-22: Rabin R, & de Charro F (2001). EQ-5D: A measure of health status from the EuroQol Group. Annals of Medicine 33(5):337-343.

Please return your completed survey in the envelope provided.

If your envelope is missing, please mail your survey to:

Survey Research Center
Harwick 7
200 first street SW
Rochester MN 55905

©2023, Mayo Foundation for Medical Education and Research (MFMER). All rights reserved.
