# Supplementary material for: Individual-Level Digital Determinants of Health and Technology Acceptance of Patient Portals: Cross-Sectional Assessment
Source: JMIR Form Res. 2024 Jun 10;8:e56493. doi: 10.2196/56493 (PMC11196914; doi:10.2196/56493)
Supplement: Multimedia Appendix 3 [file formative_v8i1e56493_app3.pdf]

Encuesta de inclusión en salud digital

Place barcode label here.

INSTRUCCIONES DE MARCADO

- Use un lápiz No 2 o un bolígrafo de tinta azul o negra solamente.
- Haz marcas sólidas que llenen la respuesta por completo.
- Rellenar el óvalo completamente.
- No haga ninguna marca fuera de las casillas.

CORRECTO: **INCORRECTO:**

PROPORCIONE POR FAVOR

Su nombre (imprima por favor):

Fecha de hoy: __ __ – __ __ – 2 0 __ __

Mes Día Año

NO ESCRIBA EN ESTA ÁREA, POR FAVOR

[SERIAL]

Las siguientes preguntas abordan el uso de computadoras (como computadoras de escritorio), teléfonos inteligentes e internet.

1. ¿Alguna vez se conecta a internet para navegar por la web o para enviar y recibir correos electrónicos?

- ⬭ Sí
- ⬭ No
- ⬭ No lo sé

2. Indique si tiene acceso a los siguientes dispositivos: (seleccione todas las opciones que correspondan)

- ⬭ Una computadora personal de escritorio o portátil
- ⬭ Una tableta, como un iPad, Samsung Galaxy, Motorola Xoom, o Kindle Fire
- ⬭ Un teléfono inteligente, como un iPhone, Android, Blackberry, o Windows Phone
- ⬭ Ninguna de las anteriores

3. ¿Accede a internet desde su casa?

- ⬭ Sí
- ⬭ No
- ⬭ No lo sé

4. ¿Está satisfecho con su acceso a internet?

- ⬭ Satisfecho
- ⬭ Ni satisfecho ni insatisfecho
- ⬭ Insatisfecho

5. Seleccione Sí, No o No lo sé para las siguientes afirmaciones.

	Si	No	No lo sé
Cuando compro un dispositivo electrónico, por lo general, necesito que otra persona lo configure o me enseñe a usarlo.	⬭	⬭	⬭
Entre mis amigos y compañeros, suelo ser el primero en probar las nuevas tecnologías de la información.	⬭	⬭	⬭
Si tuviese que enviar un mensaje a mi médico hoy, podría hacerlo sin problemas.	⬭	⬭	⬭
Si tuviese que usar una cámara o video para tener una consulta con mi médico hoy, podría hacerlo sin problemas.	⬭	⬭	⬭
He usado una tableta o un teléfono inteligente para alcanzar ciertas metas relacionadas con mi salud, como dejar de fumar, perder peso o hacer más actividad física.	⬭	⬭	⬭
Me interesaría conocer medios alternativos o nuevos para comunicarme con mi médico. .	⬭	⬭	⬭

Nos gustaría pedirle su opinión y conocer su experiencia en cuanto al uso de internet para obtener información sobre salud. Seleccione la respuesta que mejor refleje su opinión y experiencia actuales para cada una de las siguientes afirmaciones.

6. ¿Cuán útil le parece internet a la hora de tomar decisiones sobre su salud?

- ⬭ Nada útil
- ⬭ No muy útil
- ⬭ No estoy seguro
- ⬭ Útil
- ⬭ Muy útil

7. ¿Cuán importante es para usted poder acceder a recursos de salud en internet?

- ⬭ Nada importante
- ⬭ No muy importante
- ⬭ No estoy seguro
- ⬭ Importante
- ⬭ Muy importante

8. Seleccione Sí, No o No lo sé para las siguientes afirmaciones.

	Si	No	No lo sé
Sé qué recursos de salud están disponibles en internet.	⬭	⬭	⬭
Sé dónde encontrar recursos de salud útiles en internet.	⬭	⬭	⬭
Sé cómo encontrar recursos de salud útiles en internet.	⬭	⬭	⬭
Sé cómo usar internet para encontrar respuesta a mis dudas sobre la salud.	⬭	⬭	⬭
Sé cómo usar la información sobre salud que encuentro en internet para ayudarme en lo que necesito.	⬭	⬭	⬭
Tengo la capacidad para evaluar los recursos de salud que encuentro en internet.	⬭	⬭	⬭
Puedo distinguir entre recursos de salud de buena calidad y de mala calidad en internet. . . .	⬭	⬭	⬭
Me siento seguro al usar la información en internet para tomar decisiones sobre mi salud. . .	⬭	⬭	⬭

Las siguientes preguntas abordan el uso del portal para pacientes de Mayo Clinic. El portal para pacientes es una plataforma que le permite acceder a los recursos y las herramientas de Mayo Clinic desde un sitio web o una aplicación para teléfonos o tabletas.

9. ¿Su proveedor de atención médica <u>alguna vez</u> le ofreció acceso en línea a su expediente médico?

- ⬭ Si
- ⬭ No
- ⬭ No lo sé

10. ¿Alguna vez <u>tuvo</u> una cuenta en el portal para pacientes de Mayo Clinic?

- ⬭ Si
- ⬭ No
- ⬭ No lo sé

NO ESCRIBA EN ESTA ÁREA, POR FAVOR

[SERIAL]

11. ¿Tiene actualmente acceso a una cuenta en el portal para pacientes de Mayo Clinic?

- ⬭ Si → (go to 12)
- ⬭ No / ⬭ No lo sé → (go to 15)

12. ¿Alguna vez accedió a su cuenta en el portal para pacientes de Mayo Clinic?

⬭ Si ⬭ No ⬭ No lo sé

13. ¿Dejó de usar el portal para pacientes de Mayo Clinic por alguna razón en particular?

⬭ Si ⬭ No ⬭ No lo sé

14. ¿Por qué dejó de usar el portal para pacientes de Mayo Clinic? (seleccione todas las opciones que correspondan)

- ⬭ Prefiero hablar con el profesional de la salud directamente.
- ⬭ No tengo forma de acceder al sitio web.
- ⬭ No necesito consultar mi expediente médico en línea.
- ⬭ Me preocupaba la privacidad o la seguridad del sitio web donde se encontraba mi expediente médico.
- ⬭ Me resulta difícil acceder al portal para pacientes (contraseñas, dispositivos, etc.).
- ⬭ Indique otras razones por las que no usa el portal para pacientes de Mayo Clinic.

____

____

____

____

15. ¿Estaría interesado en tener acceso a una cuenta en el portal para pacientes de Mayo Clinic?

⬭ Si ⬭ No ⬭ No lo sé

16. ¿Alguna vez tuvo una cuenta en el portal para pacientes de Mayo Clinic, pero decidió dejar de usarla?

⬭ Si ⬭ No ⬭ No lo sé

17. ¿Por qué no ha usado el portal para pacientes de Mayo Clinic? (seleccione todas las opciones que correspondan)

- ⬭ Prefiero hablar con el profesional de la salud directamente.
- ⬭ No tengo forma de acceder al sitio web.
- ⬭ No necesito consultar mi expediente médico en línea.
- ⬭ Me preocupaba la privacidad o la seguridad del sitio web donde se encontraba mi expediente médico.
- ⬭ Me resulta difícil acceder al portal para pacientes (contraseñas, dispositivos, etc.).
- ⬭ Indique otras razones por las que no usa el portal para pacientes de Mayo Clinic.

____

____

____

____

18. ¿Cuán importante diría que es para usted usar el portal del paciente?

⬭	⬭	⬭	⬭	⬭	⬭	⬭	⬭	⬭	⬭	⬭
0	1	2	3	4	5	6	7	8	9	10
Nada importante										Extremadamente importante

19. Las siguientes preguntas se refieren a la facilidad de uso del portal del paciente. Indique su nivel de conformidad con las siguientes afirmaciones.1

	De acuerdo	Ni de acuerdo ni en desacuerdo	En desacuerdo
Usar el portal para pacientes exige mucho esfuerzo mental.	⬭	⬭	⬭
Usar el portal para pacientes es frustrante.	⬭	⬭	⬭
En general, creo que el portal para pacientes será fácil de usar.	⬭	⬭	⬭

20. Las siguientes preguntas abordan el apoyo que tiene en casa a la hora de usar el portal para pacientes. Indique su nivel de conformidad con las siguientes afirmaciones.1

	De acuerdo	Ni de acuerdo ni en desacuerdo	En desacuerdo
Cuento con alguien que me anima a solicitar asistencia médica a través del portal para pacientes.	⬭	⬭	⬭
Cuento con alguien a quien puedo recurrir si necesito ayuda para acceder al portal para pacientes.	⬭	⬭	⬭

21. Las siguientes preguntas abordan la utilidad de las herramientas que incluye el portal para pacientes. ¿Cuán útiles son las siguientes funciones del portal para pacientes?1

	Nada útil	No muy útil	No estoy seguro	Útil	Muy útil
Comunicación por mensajes con los proveedores de atención médica.	⬭	⬭	⬭	⬭	⬭
Programación de citas médicas.	⬭	⬭	⬭	⬭	⬭
Reposición de los medicamentos recetados.	⬭	⬭	⬭	⬭	⬭
Acceso a mi información siempre que lo desee o necesite.	⬭	⬭	⬭	⬭	⬭
Visualización de los resultados de mis pruebas.	⬭	⬭	⬭	⬭	⬭
Revisión de mis registros de vacunación.	⬭	⬭	⬭	⬭	⬭
Consultar mis síntomas.	⬭	⬭	⬭	⬭	⬭
Acceso a mis facturas.	⬭	⬭	⬭	⬭	⬭

22. Las siguientes preguntas abordan su intención de usar el portal para pacientes de Mayo Clinic más adelante. Indique su nivel de conformidad con las siguientes afirmaciones.

	De acuerdo	Ni de acuerdo ni en desacuerdo	En desacuerdo
Pretendo usar el portal para pacientes la próxima vez que necesite comunicarme con mi proveedor de atención médica.	⬭	⬭	⬭
Usaré el portal para pacientes para programar una cita con mi proveedor de atención médica.	⬭	⬭	⬭
Usaré el portal para pacientes para reponer mis recetas médicas.	⬭	⬭	⬭
Usaré el portal para pacientes cuando necesite acceder a mi información de salud.	⬭	⬭	⬭
Usaré el portal para pacientes para consultar los resultados de mis pruebas.	⬭	⬭	⬭
Usaré el portal para pacientes para consultar el material informativo relacionado con mi salud que me envíe el médico.	⬭	⬭	⬭
Usaré el portal para pacientes por alguna otra razón.	⬭	⬭	⬭

Las siguientes preguntas abordan su salud y sus capacidades en general. Debajo de cada encabezamiento, marque UNA casilla, la que mejor describe su salud HOY.

23. Movilidad2

- ⬭ No tengo problemas para caminar
- ⬭ Tengo problemas leves para caminar
- ⬭ Tengo problemas moderados para caminar
- ⬭ Tengo problemas graves para caminar
- ⬭ No puedo caminar

24. Cuidado Personal2

- ⬭ No tengo problemas para lavarme o vestirme solo/a
- ⬭ Tengo problemas leves para lavarme o vestirme solo/a
- ⬭ Tengo problemas moderados para lavarme o vestirme solo/a
- ⬭ Tengo problemas graves para lavarme o vestirme solo/a
- ⬭ No puedo lavarme o vestirme solo/a

25. Actividades de todos los días (Ej.: trabajar, estudiar, hacer las tareas domésticas, actividades familiares o actividades de ocio)2

- ⬭ No tengo problemas para realizar mis actividades de todos los días
- ⬭ Tengo problemas leves para realizar mis actividades de todos los días
- ⬭ Tengo problemas moderados para realizar mis actividades de todos los días
- ⬭ Tengo problemas graves para realizar mis actividades de todos los días
- ⬭ No puedo realizar mis actividades de todos los días

26. Dolor/Malestar2

- ⬭ No tengo dolor ni malestar
- ⬭ Tengo dolor o malestar leve
- ⬭ Tengo dolor o malestar moderado
- ⬭ Tengo dolor o malestar intenso
- ⬭ Tengo dolor o malestar extremo

27. Ansiedad/Depresión2

- ⬭ No estoy ansioso/a ni deprimido/a
- ⬭ Estoy levemente ansioso/a o deprimido/a
- ⬭ Estoy moderadamente ansioso/a o deprimido/a
- ⬭ Estoy muy ansioso/a o deprimido/a
- ⬭ Estoy extremadamente ansioso/a o deprimido/a

28. Nos gustaría saber lo buena o mala que es su salud HOY. La escala está numerada de 0 a 100. 100 representa la mejor salud que se pueda imaginar. 0 representa la peor salud que se pueda imaginar. Por favor, indique en esta escala cómo es su salud HOY.2

Su Salud Hoy= [ ][ ][ ]

La peor salud que se pueda imaginar — La mejor salud que se pueda imaginar

0 5 10 15 20 25 30 35 40 45 50 55 60 65 70 75 80 85 90 95 100

29. En general, diría que su salud es:

⬭ Excelente ⬭ Muy buena ⬭ Buena ⬭ Regular ⬭ Mala

30. Tengo una buena relación con las personas que me brindan atención médica.

⬭ De acuerdo ⬭ Ni de acuerdo ni en desacuerdo ⬭ En desacuerdo

31. Pienso que mi profesional de la salud quiere lo mejor para mí.

⬭ De acuerdo ⬭ Ni de acuerdo ni en desacuerdo ⬭ En desacuerdo

32. **¿Alguna vez un médico, un miembro del personal de enfermería o de enfermería especializada, u otro profesional de la salud le preguntó sobre su creencias culturales o religiosas en relación con su salud?**

- ⬭ Sí
- ⬭ No
- ⬭ No lo sé

Información demográfica

33. **¿Con qué identidad de género se identifica más?**

- ⬭ Mujer
- ⬭ Hombre
- ⬭ Mujer transgénero
- ⬭ Hombre transgénero
- ⬭ Género no binario / no conforme
- ⬭ Prefiero no responder
- ⬭ Si su identidad no figura aquí, especifique: ______________________________

34. **¿Con qué raza o etnia se identifica más?** (seleccione todas las opciones que correspondan)

- ⬭ Asiática, Surasiática o Asiática del Pacífico
- ⬭ Negra, Africana o Afroamericana
- ⬭ Centroamericana o Sudamericana
- ⬭ Mexicana o Mexicoamericana
- ⬭ Medioriental
- ⬭ Indígena Estadounidense
- ⬭ Hawaiana o de otras islas del Pacífico
- ⬭ Blanco Americano, Caucásico o Blanco Europeo
- ⬭ Me identifico con más de una raza
- ⬭ Ninguna de las anteriores
- ⬭ Prefiero no responder
- ⬭ Ninguna de las anteriores: ______________________________

35. **¿En qué idioma le resulta más cómodo leer?** (seleccione todas las opciones que correspondan)

- ⬭ Árabe
- ⬭ Cantonés
- ⬭ Inglés
- ⬭ Farsi
- ⬭ Hindi
- ⬭ Mandarín
- ⬭ Español
- ⬭ Somalí
- ⬭ Urdu
- ⬭ Prefiero no responder
- ⬭ Si su idioma no figura aquí, especifique: ______________________________

36. **¿Cuán cómodo se siente leyendo en Inglés?**

- ⬭ Incómodo
- ⬭ Ni incómodo ni cómodo
- ⬭ Cómodo

37. **¿Cuán cómodo se siente hablando en Inglés?**

- ⬭ Incómodo
- ⬭ Ni incómodo ni cómodo
- ⬭ Cómodo

38. **Por favor, informe al equipo de investigación de cualquier otro pensamiento o comentario que tenga relacionado con este tema:**

____

____

____

¡Gracias por completar esta encuesta!

Questions 13-15: 1Emani S, Peters E, Desai S, Karson AS, Lipsitz SR, LaRocca R, Stone J, Suric V, Wald JS, Wheeler A, Williams DH, Bates DW. Who adopts a patient portal?: An application of the diffusion of innovation model. J Innov Health Inform. 2018 Oct 25;25(3):149-157. doi: 10.14236/jhi.v25i3.991. PMID: 30398458.

Questions 17-22: 2Rabin R, & de Charro F (2001). EQ-5D: A measure of health status from the EuroQol Group. Annals of Medicine 33(5):337-343.

Devuelva su encuesta completa en el sobre provisto, por favor. Si falta su sobre, envíe su encuesta por correo a:

Survey Research Center
Harwick 7
200 first street SW
Rochester MN 55905

©2023, Fundación Mayo para la Educación e Investigación Médicas (MFMER). Todos los derechos reservados.

NO ESCRIBA EN ESTA ÁREA, POR FAVOR

[SERIAL]
