# Supplementary material for: Individual-Level Digital Determinants of Health and Technology Acceptance of Patient Portals: Cross-Sectional Assessment
Source: JMIR Form Res. 2024 Jun 10;8:e56493. doi: 10.2196/56493 (PMC11196914; doi:10.2196/56493)
Supplement: Multimedia Appendix 4 [file formative_v8i1e56493_app4.docx]

Descriptive comparisons of characteristics among responders and nonresponders was done using chi-square for gender and race, and t-test for age. There was a significant difference in response by age (responders were older) and across race groups (responders were more often White). There was no significant difference in gender.

Characteristics of responders’ vs non-responders

| Demographics |  | |
| --- | --- | --- |
|  | Responders | Non-responders |
| N=11424 | 1850 | 9574 |
| Gender:  Female  Male  Unknown | 1078 (58.3%)  772 (41.7%)  0 (0.0%) | 5555 (58.0%)  4016 (41.9%)  3 (0.0%) |
| Race*:  Asian  Black  Native American  Other  White | 130 (7.0%)  102 (5.5%)  42 (2.3%)  148 (8.0%)  1428 (77.2%) | 823 (8.6%)  996 (10.4%)  256 (2.7%)  1487 (15.5%)  6012 (62.8%) |
| Age*: Mean (SD) | 63.3 (14.7) | 52.5 (17.8) |

*significant differences between responders and nonresponders

When comparing response rates by language, we found a higher response rate among people who had declared English as their primary language and received the English language survey, compared those who declared Spanish as their primary language and received the Spanish language survey (18.6% English vs 8.1% Spanish).

|  | English survey | | | | Spanish survey | | | |
| --- | --- | --- | --- | --- | --- | --- | --- | --- |
|  | Total=8795 | | | | Total=2629 | | | |
|  | Responders | Response Rate | Non-responders | Non-response Rate | Responders | Response Rate | Non-responders | Non-response Rate |
| Response Type | 1638 | 0.186242 | 7157 | 0.81375782 | 212 | 0.080639 | 2417 | 0.91936097 |
